# Coenzyme Q_2_ is a universal substrate for the measurement of respiratory chain enzyme activities in trypanosomatids

**DOI:** 10.1051/parasite/2019017

**Published:** 2019-03-22

**Authors:** Petra Čermáková, Tomáš Kovalinka, Kristína Ferenczyová, Anton Horváth

**Affiliations:** 1 Department of Biochemistry, Faculty of Natural Sciences, Comenius University Bratislava Slovakia

**Keywords:** ubiquinone, respiratory chain, Coenzyme Q

## Abstract

The measurement of respiratory chain enzyme activities is an integral part of basic research as well as for specialized examinations in clinical biochemistry. Most of the enzymes use ubiquinone as one of their substrates. For current *in vitro* measurements, several hydrophilic analogues of native ubiquinone are used depending on the enzyme and the workplace. We tested five readily available commercial analogues and we showed that Coenzyme Q_2_ is the most suitable for the measurement of all tested enzyme activities. Use of a single substrate in all laboratories for several respiratory chain enzymes will improve our ability to compare data, in addition to simplifying the stock of chemicals required for this type of research.

## Abstract (fr)

La mesure des activités des enzymes de la chaîne respiratoire fait partie intégrante de la recherche fondamentale ainsi que des examens spécialisés en biochimie clinique. La plupart des enzymes utilisent l’ubiquinone comme l’un de leurs substrats. Pour les mesures *in vitro* actuelles, plusieurs analogues hydrophiles de l’ubiquinone native sont utilisés en fonction de l’enzyme et du lieu de travail. Nous avons testé cinq analogues commerciaux facilement disponibles et avons montré que la coenzyme Q_2_ était la mieux adaptée à la mesure de toutes les activités enzymatiques testées. L’utilisation d’un seul substrat dans tous les laboratoires pour plusieurs enzymes de la chaîne respiratoire améliorera notre capacité à comparer les données, tout en simplifiant le stock de produits chimiques nécessaires à ce type de recherche.

## Introduction

Trypanosomatids are obligatory parasites belonging to the Family Trypanosomatidae and Phylum Euglenozoa. Many representatives of this group that change between two hosts – insect vectors and higher animals or plants – are the cause of serious diseases in humans, animals and plants (e.g. sleeping sickness in Africa, Chagas disease in Latin America, and various types leishmaniases worldwide). In addition to these dixenic species, many new monoxenic species have been isolated in recent years and have only one host – insects. Major differences in their mitochondrial metabolism [20, 24] indicate that further study could be positive for both basic and applied research. The respiratory chain (RC) is the central and essential part of the mitochondrial bioenergetics of the cell and its disorders are associated with multiple metabolic diseases [13]. Therefore, measuring the activity of the RC enzymes will bring new knowledge to basic and medical research. It could potentially also help in the development of treatments for diseases caused by trypanosomatids. The RC consists of four so-called “core” enzyme complexes I–IV. Three of them (Complex I – NADH dehydrogenase, Complex III – cytochrome *c* reductase, and Complex IV – cytochrome *c* oxidase) use the energy of transferred electrons to create a proton gradient on the inner mitochondrial membrane, which is further utilised in ATP biosynthesis. Complex II – succinate dehydrogenase is an integral component of both the Krebs cycle and the RC, but it does not contribute to membrane potential. In addition to the core enzymes, the RC of many cells also includes several so-called alternative enzymes that transmit electrons within the RC, but without pumping protons across the inner mitochondrial membrane: for example, alternative NADH dehydrogenase (NDH2) acting in parallel with Complex I, or Trypanosome alternative oxidase (TAO) bypassing complexes III and IV. Furthermore, two low-molecular mass compounds participate in the transfer of electrons in the RC: cytochrome *c* and ubiquinone. The second compound belongs to a group of chemical compounds containing a quinoid ring system that can exist in several states depending on the presence or absence of electrons. Ubiquinone can be found in three different oxidation-reduction states: (i) fully oxidized form – ubiquinone, (ii) partially reduced and unstable semiquinone, created by the receipt of one electron, and (iii) fully reduced ubiquinol (hydroubiquinone) with two accepted electrons. Ubiquinone, which is an integral part of the RC is also called Coenzyme Q_10_ (Q_10_). The digit in its name is originally derived from number of isoprenyl subunits in its side chain; however, despite the number 10, their number in different organisms varies from 6 to 10 [27]. Q_10_ is a substrate for most of the enzymes involved in electron transfer within the inner mitochondrial membrane. Some enzymes use the oxidised form of ubiquinone as an electron acceptor (e.g. both NADH dehydrogenases and succinate dehydrogenase), whereas others use its reduced form (ubiquinol) as an electron source (cytochrome *c* reductase and alternative oxidase). However, the hydrophobic tail of Q_10_ makes this compound unsuitable for *in vitro* experiments due to very low solubility in aqueous solutions. That is the reason why various Q_10_ analogues are used for the *in vitro* measurement of RC enzyme activities. The literature describes the use of different Q coenzymes for measuring RC enzyme activities; they even vary for individual enzymes. For example, NADH dehydrogenase is measured with Coenzyme Q_1_ (Q_1_) [2], Coenzyme Q_2_ (Q_2_) [7, 24] and Decylubiquinone (DB) [26]; succinate dehydrogenase with Q_1_ [2] and Q_2_ [2, 24]; cytochrome *c* reductase with either reduced Coenzyme Q_2_ (Q_2_H) [2] or with reduced Decylubiquinone (DBH) [10, 22]; and the alternative oxidase with reduced Coenzyme Q_1_ (Q_1_H) and Q_2_H [12, 17]. To allow comparability of the results obtained from different laboratories and to simplify stocks of chemicals needed for the assays, we tested the suitability of five commercially available ubiquinone analogues for the measurement of RC enzyme activities.

We used three different species of trypanosomatids as models. They differ in both the composition of their respiratory chain, and the strength of the activities of individual components. This makes them a suitable model for the standardisation of enzyme activity measurement. *Phytomonas serpens* has lost two “core” respiratory chain enzymes – cytochrome *c* reductase and cytochrome *c* oxidase [15]. *Trypanosoma brucei* (TB) does not have a fully functional complex I in the procyclic life stage (TB (PF)) [23], which dramatically lowers the activity of the respiratory chain. Reduced ubiquinone is regenerated only by TAO in the blood stream form (TB(BF)) because activity of the rest of the respiratory chain is reduced in this cell cycle stage (for review see [21]). *Leishmania tarentolae* has no TAO and no measurable NADH dehydrogenase activity [16, 18, 24]. Coenzyme Q_2_ proved to be the optimal substrate for all tested enzymes in trypanosomatids. The universality of the presented results was demonstrated by the use of Coenzyme Q_2_ to measure respiratory chain enzyme activities in mitochondrial lysates of a yeast (*Saccharomyces cerevisiae*) and a vertebrate (chicken liver).

## Materials and methods

### Tested coenzymes

Coenzyme Q_1_ (C7956, 2,3-Dimethoxy-5-methyl-6-(3-methyl-2-butenyl)-1,4-benzoquinone, Ubiquinone-1, Ubiquinone-5, C_14_H_18_O_4_); Coenzyme Q_2_, **(**C8081, 2,3-Dimethoxy-5-methyl-6-geranyl-1,4-benzoquinone, Ubiquinone-10, C_19_H_26_O_4_); Decylubiquinone, (D7911, 2,3-Dimethoxy-5-methyl-6-decyl-1,4-benzoquinone, C_19_H_30_O_4_); Coenzyme Q_4_ (C2470, 2,3-Dimethoxy-5-methyl-6-(geranylgeranyl)-1,4-benzoquinone, Q-4, Ubiquinone-20, Ubiquinone-4, C_29_H_42_O_4_**)** and Coenzyme Q_10_ (C9538, Q-10, Ubiquinone 50, Ubiquinone-10, C_59_H_90_O_4_) were purchased from Sigma-Aldrich. The chemical names of ubiquinones often do not correspond to their commercial names as coenzymes. Therefore, to avoid possible misunderstandings, we use the term coenzyme Q as far as possible.

### Cultivation of trypanosomatids

The procyclic form of *Trypanosoma brucei* (strain 29–13) was cultivated in regular SDM-79 supplemented with 10% (v/v) heat-inactivated fetal bovine serum (FBS), as previously described [25]. *Leishmania tarentolae* (strain UC) and *Phytomonas serpens* (strain 9T) were grown in BHI medium supplemented with 10 μg/mL hemin [14]. Cultures were kept at 27 °C and diluted 10× upon reaching 1 × 10^7^/mL for *T. brucei* and 5 × 10^7^/mL for the other species. The bloodstream form of *Trypanosoma brucei* was cultured at 37 °C with 5% CO_2_ in HMI-9 medium supplemented with 3 g/L sodium bicarbonate, 136 mg/L hypoxanthine, 110 mg/L pyruvate, 39 mg/L thymidine, 28 mg/L bathocuproinedisulfonic acid, 182 mg/L l-cysteine, 0.0014% (v/v) 2-mercaptoethanol, 10% (v/v) heat-inactivated FBS, 100 unit/mL penicillin and 0.1 mg/mL streptomycin [19].

### Preparation of mitochondrial lysate

Mitochondria-enriched fractions were obtained as described previously [11]. Next, mitochondria were suspended in 0.5 M aminocaproic acid and 10% (w/v) dodecyl maltoside was added to a final concentration of 2% (w/v). Lysis was performed for 60 min on ice and the lysate was centrifuged for 10 min at maximum speed at 4 °C. The supernatant was recovered and protein concentration was determined by the Bradford assay [3].

### Coenzyme reduction

The reduced form of coenzyme (ubiquinol) was prepared by reduction of appropriate ubiquinone (Coenzymes Q_1_, Q_2_, Q_4_, Q_10_ and DB) using a procedure adapted from [22]. Coenzyme was diluted in acidic ethanol (96% (v/v) ethanol, 1 mM acetic acid) to the final concentration 25 mM. One mL of this solution was mixed with 1 mL of 500 mM NaPi, pH 7.4 and 3 μL of 1 M HCl and the solution was sparged with nitrogen to remove oxygen. Then, 13 mg of sodium dithionite and 1 μL of 1 M HCl were added. After a short vortex, 13 mg of sodium borohydride were added. The resulting colourless solution was extracted three times with 3 mL of cyclohexane and incubated 1–2 h with 200 mg of sodium sulfate to absorb the remaining water in the organic phase. The solution was transferred to a new tube, and the cyclohexane was evaporated by nitrogen sparging. The reduced coenzyme was dissolved in acidic ethanol to the final concentration 10 mM, split into small aliquots, and stored under nitrogen at −80 °C until required.

Coenzymes Q_1_ and Q_2_ (but not Q_4_, Q_10_ and DB) could also be reduced by a simpler alternative method adapted from [2]. A 2 mL solution of coenzyme in acidic ethanol, NaPi and HCl was directly mixed with 2 mL of cyclohexane, and 20–30 mg of sodium dithionite was added. The tube was thoroughly mixed by vortex until the solution became colourless. The organic phase was removed, and the extraction was repeated twice. Collected cyclohexane containing reduced coenzyme was evaporated, as described above.

### Enzymatic assays

NADH dehydrogenase activities were measured as previously described [7] with some modifications. Briefly, 5 μL of mitochondrial lysate and 5 μL of 20 mM NADH were mixed with 1 mL of NDH buffer (50 mM KPi pH 7.5; 1 mM EDTA, pH 8.5; 0.2 mM KCN). The reaction was started by the addition of 2 μL of 10 mM tested coenzyme. The reaction was followed at 340 nm for 3 min.

Succinate dehydrogenase was measured as previously described [2] with some modifications. Briefly, 5 μL of the mitochondrial lysate was mixed with 1 mL of SDH buffer (25 mM KPi, pH 7.2; 5 mM MgCl_2_; 20 mM sodium succinate) and incubated in 30 °C for 10 min. Next, antimycin A, rotenone, KCN and 2,6-dichlorophenolindophenol were separately added to a final concentration of 2 μg/mL, 2 μg/mL, 2 mM and 50 μM, respectively, and then mixed together. The reaction background was monitored at 600 nm for 5 min and its value was subtracted from measured activity. The reaction itself was started by adding of tested coenzyme to a final concentration of 65 μM, and was followed at 600 nm for 5 min.

The activity of cytochrome *c* reductase was measured as previously described [10] with minor modifications. Simultaneously, 2 μL of the mitochondrial lysate and 2 μL of 10 mM reduced tested coenzyme were added to 1 mL of QCR buffer (40 mM NaPi, pH 7.4; 0.5 mM EDTA; 20 mM sodium malonate; 50 μM cytochrome *c*; 0.005% (w/v) dodecyl maltoside) and briefly mixed. The reaction was monitored at 550 nm for 1 min.

Alternative oxidase was measured as previously described [12]. Briefly, 5 μL of mitochondrial lysate was added to 1 mL of 50 mM Tris–HCL (pH 7.4) and incubated in 25 °C for 2 min. The reaction was initiated by the addition of reduced tested coenzyme to a final concentration of 150 μM, and was followed at 278 nm for 5 min.

Protein concentrations of mitochondrial lysates were about 8 mg/mL (±2.5 mg/mL) and the measured activity was converted to 1 mg of proteins. All inhibitor solutions were freshly prepared. Rotenone was dissolved in dimethylsulfoxide, DPI in methanol, sodium malonate in water, and antimycin A and salicylhydroxamic acid (SHAM) in ethanol. Inhibitors were added to the assay mixture immediately before the start of the reaction in the concentrations listed in Table 1.

**Table 1:** Respiratory chain enzyme activities with different substrates.

NADH dehydrogenase		Activity [U/mg]	SD	Inh. DPI %	Inh. Rot. %	Cytochrome *c* reductase		Activity [mU/mg]	SD	Inh. Ant. %
*P. serpens*	Q_1_	0	0	–	–	*T. brucei* (PF)	Q_1_H	432	98	27
	Q_2_	53	25	37	36		Q_2_H	383	124	86
	DB	45	4	0	17		DBH	410	101	95
*T. brucei* (PF)	Q_1_	65	23	100	–	*L. tarentolae*	Q_1_H	609	19	19
	Q_2_	81	19	74	–		Q_2_H	611	118	83
	DB	43	29	2	–		DBH	755	151	96
										
Succinate dehydrogenase		Activity [U/mg]	SD	Inh. Mal. %		Alternative oxidase		Activity [U/mg]	SD	Inh. SHAM %
*P. serpens*	Q_1_	20	11	94		*P. serpens*	Q_1_H	398	120	89
	Q_2_	24	10	100			Q_2_H	538	71	79
	DB	19	4	100			DBH	105	5	92
*T. brucei* (PF)	Q_1_	45	12	99		*T. brucei* (PF)	Q_1_H	8	3	7
	Q_2_	54	14	98			Q_2_H	10	4	84
	DB	27	12	100			DBH	0	0	–
*L. tarentolae*	Q_1_	104	28	99		*T. brucei* (BF)	Q_1_H	146	51	84
	Q_2_	122	18	99			Q_2_H	282	83	95
	DB	86	11	100			DBH	52	1	0

Average values of enzyme activities are displayed in Figure 2 where activities [U] of individual enzymes are also defined; SD – standard deviations; Inh. – the rate of inhibition with the corresponding inhibitor in %; DPI – diphenyl iodonium (150 μM); Rot. – rotenone (10 μM); Ant. – Antimycin A (150 μM); Mal. – sodium malonate (1 mM); SHAM – salicylhydroxamic acid (10 μM); Inhibition with rotenone was not measured with *T. brucei*, because it was already shown previously that NADH dehydrogenase activity in this organism is not sensitive to rotenone [23].

## Results and discussion

**Figure 1:**
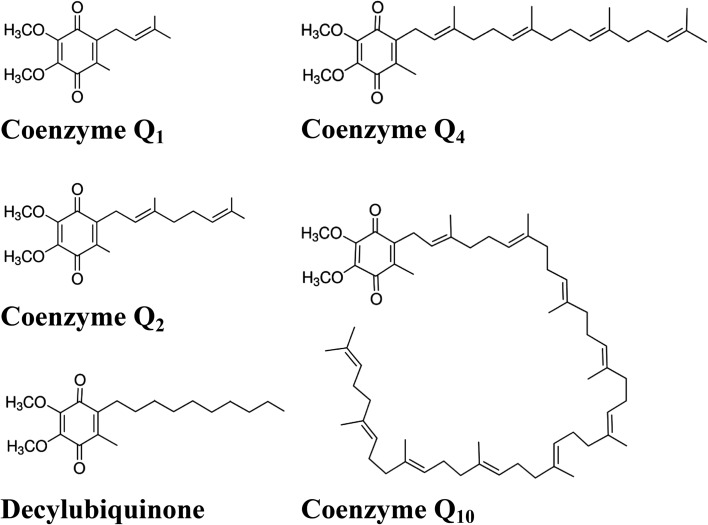
Chemical structures of the tested coenzymes.

**Figure 2:**
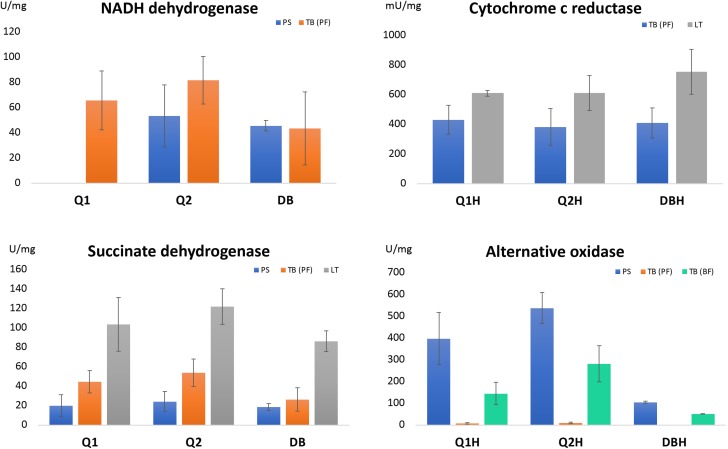
Respiratory chain enzyme activities with different substrates. PS – *Phytomonas serpens*, TB(PF) – *Trypanosoma brucei* (procyclic form), TB(BF) – *Trypanosoma brucei* (blood stream form), LT – *Leishmania tarentolae*. The unit (U) of appropriate activity is defined as an amount of enzyme required for conversion of: 1 nMol of NADH/min for NADH dehydrogenase; 1 nMol of 2,6-dichlorophenolindophenol/min for succinate dehydrogenase; 1 μMol of cytochrome *c* for cytochrome *c* reductase and 1 nMol of appropriate coenzyme Q/min for TAO. Presented data are an average of at least three independent biological experiments, each measured in triplicate. Only trypanosomatids with measurable corresponding activity in their mitochondria are shown.

The respiratory chain in all three trypanosomatids has already been investigated using different Q coenzymes to measure individual enzyme activities. While NADH dehydrogenase, succinate dehydrogenase and cytochrome *c* reductase were experimentally tested in all three species, TAO activity was only measured in the case of heterologous expression of the respective *T. brucei* gene in *E. coli* [12, 17], and its presence was indirectly proven in *P. serpens* by measurement of oxygen consumption sensitive to TAO-specific inhibitor [24]. In this study, we tested five Q coenzymes that differ in their side chain. However, the length or character of its hydrophobic tail can influence its interaction with the particular examined enzyme [9]. Therefore, we tested with all substrates not only the absolute activity of the examined enzymes, but also the sensitivity of the measured activity to inhibitors specific to individual enzymes: rotenone – Complex I, diphenyl iodonium (DPI) – NDH2, sodium malonate – Complex II, antimycin A – Complex III and SHAM – TAO. We used assays that have already been published to measure each activity. Therefore, we assumed that each individual method was already optimised. Our goal was to test whether we could obtain comparable or better measurable values with substrates other than those that have been used so far. We confirmed that Q_10_ is not suitable for the *in vitro* measurement of any tested enzyme activity. Similarly, Q_4_ was not usable either. Obtained activities with these two substrates were not measureable, or were substantially lower (between 5- and 30-fold) than with the other three coenzymes. The probable reason for both compounds is the high hydrophobicity of their aliphatic chain (see Fig. 1). Therefore, we performed a full set of measurements only with Q_1_, Q_2_ and DB (see Fig. 2 and Table 1) and the indicative activity values with Q_4_ and Q_10_ are given only in the text. We used two different methods to reduce all three coenzymes. While a longer method adapted from [22] totally reduced all three tested coenzymes, the simpler method described by [2] sufficiently reduced only Q_1_ and Q_2_. DBH reduced by this method was not a suitable substrate for these measurements.

### NADH dehydrogenase

NADH dehydrogenase activity in trypanosomatid mitochondria could correspond to two different enzymes: Complex I and NDH2. While Complex I is sensitive to rotenone [8], NDH2 is rotenone-resistant and is sensitive to DPI at concentrations that do not influence Complex I [1, 4, 6]. There was almost no activity with Q_4_ for *P. serpens* (2 U) and *T. brucei* (1.5 U) and zero with Q_10_ for both examined trypanosomatids. The highest NADH dehydrogenase activity was obtained with Q_2_ as substrate (NADH dehydrogenase Fig. 2, Table 1) and absolute values, as well as sensitivity to inhibitors, are comparable with results reported in the literature [4, 24]. A high activity value in *T. brucei* was also recorded with Q_1_, however essentially no signal in *P. serpens* disqualifies this coenzyme as a universal substrate for NADH dehydrogenase. Reasonably high activity values were obtained with DB in both *P. serpens* and *T. brucei*. However, very low or no efficiency of inhibition suggest that DB is not a suitable substrate for *in vitro* NADH dehydrogenase activity measurements in trypanosomatids. Minimal sensitivity to rotenone with DB was also demonstrated for the same activity in bovine heart mitochondria [5]. NADH dehydrogenase activity has the most striking difference in relative activity with one substrate between trypanosomatids: from no activity in *P. serpens* with Q_1_, through 1.5 times lower activity in *P. serpens* than in *T. brucei* with Q_2_, and almost equal activities in both species with DB. For all other enzyme substrate combinations, the relative activities between the tested species did not differ significantly. The same is true for the sensitivity to inhibitors. This may reflect the different contribution of two enzymes (Complex I and NDH2) to total activity measured in different trypanosomatid species, or might indicate that the active sites corresponding to this activity differ more than with other enzymes between trypanosomatids.

### Succinate dehydrogenase

All three substrates Q_1_, Q_2_ and DB were suitable for measurement of succinate dehydrogenase in all tested cell lines. The activities and their relative ratio in individual strains were comparable. Nevertheless, activity with Q_2_ was the highest in all three species (Succinate dehydrogenase Fig. 2; Table 1). Inhibition with competitive inhibitor sodium malonate was more than 90% in all combination substrates and trypanosomatids. Our data suggest that succinate dehydrogenase is the enzyme with the lowest requirements to coenzyme Q specificity. Nevertheless, the activities were significantly lower with two of the most hydrophobic substrates (Q_4_: 1 U *P. serpens*, 7 U *T. brucei* and 9 U *L. tarentolae*; Q_10_: 0 U *P. serpens*, 1 U *T. brucei* and 4 U *L. tarentolae*).

### Cytochrome *c* reductase

The relevance of Q_1_H, Q_2_H and DBH for cytochrome *c* reductase resembles the situation with succinate dehydrogenase. Activities of all three substrates were comparable in both tested species. However, very low inhibition by antimycin A with Q_1_H shows that this coenzyme is not the best substrate for the enzyme (Cytochrome *c* reductase Fig. 2; Table 1). Measured activities of cytochrome *c* reductase with both Q_4_ and Q_10_ were zero. Although activity with DBH was slightly higher than Q_2_, both compounds are good substrates for the third respiratory chain enzyme. However, the reduction procedure for Q_2_H is simpler than DBH (see section Materials and Methods). This is why we again favour Q_2_H as the most suitable coenzyme for the measurement of cytochrome *c* reductase activity.

### Trypanosome alternative oxidase

TAO activity has the highest differences of absolute activities between comparable cell lines. The values of *P. serpens* are approximately two times higher than *T. brucei* (BF) and even 50 times higher than *T. brucei* (PF) (Alternative oxidase Fig. 2, Table 1). Significantly, the lowest were activities with Q_4_ (21 U with *P. serpens* and 5 U *T. brucei* (PF)) and Q_10_ (23 U *P. serpens* and 15 U *T. brucei* (PF); we did not use these two substrates to measure TAO activity in *T. brucei* (BF). Activities with DBH are remarkably lower than with the other two hydrophylic substrates (in TB (PF) cells activity was not measurable). The highest signals were again with Q_2_H, and only slightly lower with Q_1_H. Despite the fact that in the other laboratory TAO activity was measured with Q_1_H [12, 17], in TB (PF) the activity signal was not inhibited by SHAM. For this reason, we do not consider Q_1_H to be a universal substrate for this enzyme. On the basis of the obtained data, we conclude that Q_2_H is the best substrate for TAO.

### Respiratory chain enzymes of *S. cerevisiae* and chicken liver mitochondria

To verify the general validity of our results, we applied Coenzyme Q_2_ to measure activities of NADH dehydrogenase, succinate dehydrogenase and cytochrome *c* reductase in both mitochondrial lysates of *S. cerevisiae* and chicken liver (Table 2). The values of measured activities in both organisms were roughly comparable with those obtained for the trypanosomatids investigated in this study. This also confirms the suitability of Q_2_ as a substrate for the respiratory chain enzymes in these evolutionarily divergent organisms.

**Table 2:** Respiratory chain enzyme activities in *S. cerevisiae* and chicken liver.

Q_2_		Activity	SD	Inh. %
*S. cerevisiae*	NADH	404	121	59
	SDH	146	3	95
	QCR	840	20	87
Chicken liver	NADH	39	6	47
	SDH	16	1	100
	QCR	540	40	67

Activities [U] of individual enzymes are defined in Figure 2; Q_2_ – Coenzyme Q_2_ used as a substrate for activity measurement. NADH – NADH dehydrogenase; SDH – succinate dehydrogenase; QCR – cytochrome c reductase; SD – standard deviations; Inh. – the rate of inhibition with the corresponding inhibitor in %: NADH – DPI for *S. cerevisiae* which do not possess complex I and rotenone for chicken liver; SDH – sodium malonate and QCR – antimycin A.

## Conclusions

Today’s practice in measuring respiratory chain enzyme activities is that different coenzyme Q_10_ analogues are used for each enzyme. We have shown that a single variant can be used for all enzymes that use Q coenzymes as substrates. Our data clearly show that out of all the readily commercially available ubiquinones, coenzyme Q_2_ is an optimal substrate for all tested enzymes. Q_2_ is an appropriate substrate not only for trypanosomatids, but is also suitable for evolutionarily distant organisms, such as yeasts and vertebrates, thus corroborating the general validity of our conclusions.

## Note

Tomáš Kovalinka won the prize for the best student presentation at the 48th Jírovec’s Protozoological Days, held on April 30 – May 4, 2018 at Kunčice pod Ondřejníkem, Czech Republic, which was a full APC waiver for a paper in *Parasite*. The cost of publication of the present paper was thus offered by EDP Sciences.
